# Resilience of the Gut Microbiome to Short Proton Pump Inhibitor Therapy With or Without High‐Dosage *L. reuteri* in *H. pylori*‐Infected Adults

**DOI:** 10.1111/hel.70064

**Published:** 2025-09-24

**Authors:** Stefano Bibbò, Gustav Ahlström, Giovanni Mario Pes, David Y. Graham, Lars Engstrand, Elettra Merola, Maria Pina Dore

**Affiliations:** ^1^ Department of Medicine, Surgery and Pharmacy University of Sassari Sassari Italy; ^2^ Centre for Translational Microbiome Research, Department of Microbiology, Tumor and Cell Biology Karolinska Institute Solna Sweden; ^3^ Baylor College of Medicine Houston Texas USA

**Keywords:** gut microbiota, *Helicobacter pylori*, *Lactobacillus reuteri*, probiotics, proton pump inhibitors

## Abstract

**Background:**

*Helicobacter pylori* eradication therapy typically consists of a combination of antibiotics and an antisecretory drug. Probiotics may be added to reduce side effects and possibly improve outcomes.

**Materials and Methods:**

We conducted a double‐blind, randomized trial of pantoprazole plus either *Lactobacillus reuteri* (Gastrus) (high dose) or a matching placebo to assess the impact on the gut microbiota of *H. pylori* ‐positive adults. Fecal samples were collected at baseline and after one and 2 months for shotgun metagenomic sequencing.

**Results:**

A total of 26 patients were recruited and completed therapy. *L. reuteri* was only detected in the group that received supplemental *L. reuteri* and only at the 1‐month post‐treatment interval. *L. reuteri* failed to colonize for long‐term the gut, and challenge with *L. reuteri* failed to alter alpha‐diversity (Shannon index) or beta‐diversity (community ordination) metrics at any time point. Machine learning (PLS‐DA) analysis identified the presence of *L. reuteri* as the most distinguishing feature at 1 month. No other taxa showed a significant difference between groups.

**Conclusion:**

Short‐term administration of pantoprazole and *L. reuteri* had no lasting effects on gut microbial composition. While *L. reuteri* transiently bloomed during supplementation, the overall gut microbiota showed resilience, returning to baseline shortly after therapy.

**Trial Registration:**

Identifier: NCT03404440

## 1 Introduction

*Helicobacter pylori* is a highly prevalent gastric pathogen that infects roughly half of the world's population, typically acquired in childhood [1]. Its eradication usually requires a multi‐drug regimen combining antibiotics with acid suppression [2]. In recent years, probiotics have been investigated as adjuvant therapy to improve eradication rates and reduce treatment side effects [3]. A recent meta‐analysis of randomized trials involving 13,680 patients concluded that adding probiotics (especially *Bifidobacterium longum* ) to standard triple or quadruple therapy produced significant benefits, including improved eradication rates (ITT analysis: from 62% to 79%, OR = 1.62) and reduced gastrointestinal side effects [4, 5, 6, 7]. A previous meta‐analysis of randomized trials consistently showed that adding probiotics (such as Lactobacillus or Saccharomyces strains) to standard therapy yielded modest but significant gains, with eradication rates improving by roughly 10%–15%, along with a reduction in therapy‐related diarrhea and other symptoms [8, 9, 10, 11, 12, 13, 14].

Overall, probiotics are hypothesized to act by competing with pathogens for adhesion sites, modulating immune responses, and/or stabilizing the gut microbiota during antibiotic therapy. More specifically, a recent meta‐analysis of eight trials (1087 patients) reported that *L. reuteri* supplementation in eradication therapy increased *H. pylori* eradication rates by ~7% (e.g., from ~73% to ~80%) and significantly reduced therapy‐related side effects [15]. Mechanistically, *L. reuteri* is thought to act by producing antimicrobial metabolites (such as reuterin and organic acids) that inhibit *H. pylori* growth and may help rebalance the gastric environment [16]. However, probiotic effects are typically strain‐specific and often transient, as even well‐studied strains, such as *L. reuteri* , tend to dominate the gut microbiota only during the period when they are being administered. Persistent colonization after treatment is rare [17, 18].

Proton pump inhibitors (PPIs) are used to enhance the efficacy of antibiotics by raising gastric pH. However, PPIs are also known to perturb the gastrointestinal microbiota [19, 20], and mounting evidence suggests a link between chronic PPI use and gut microbial dysbiosis. For example, a meta‐analysis of sequencing data from PPI users reported a significant decrease in gut alpha diversity as well as depletion of beneficial short‐chain fatty acid‐producing taxa (e.g., *Ruminococcaceae* and *Lachnospiraceae*) compared to non‐users [21]. Hypochlorhydria induced by PPIs may allow upper gastrointestinal and oral bacteria (e.g., *Streptococcaceae*, *Actinomyces*, *Rothia*) to survive and flourish in the distal gut [21]. Correspondingly, PPI use has been associated with increased intestinal *Enterococcaceae* and *Streptococcaceae* and reductions in *Clostridiales* [22, 23]. These shifts may predispose patients to small intestinal bacterial overgrowth (SIBO) and *Clostridioides difficile* infections [24, 25]. Long‐term PPI therapy has also been epidemiologically linked to a higher incidence of enteric infections and nutrient malabsorption [26, 27]. These findings suggest that PPI‐associated dysbiosis might undermine microbiota‐mediated health and adherence during *H. pylori* therapy.

It remains unclear whether probiotic supplementation can mitigate PPI‐induced gut dysbiosis associated with *H. pylori* eradication therapy. More generally, probiotics have been shown to suppress the overgrowth of opportunistic pathogens and reduce antibiotic‐ or drug‐induced diarrhea [28]. In one pediatric study, co‐administration of *L. reuteri* with a PPI significantly reduced the incidence of post‐treatment SIBO, suggesting a beneficial protective effect on the microbiota [28].

We report the results of a single‐center, double‐blind randomized trial comparing the effects of a 4‐week regimen of pantoprazole plus high‐dose *L. reuteri* (Gastrus) versus pantoprazole plus placebo on the gut microbiota of *H. pylori* ‐infected adults. The goal was to investigate whether the addition of *L. reuteri* would result in a beneficial long‐term gut microbial profile in the presence of PPI therapy. Antibiotic therapy was intentionally omitted in this trial in order to isolate the microbiome effects of the PPI and probiotic. Standard eradication treatment was offered post‐study to ensure all patients received appropriate care.

## 2 Materials & Methods

### 2.1 Study Design and Participants

We conducted a single‐center, double‐blind, randomized, placebo‐controlled trial at the University of Sassari, Italy. Twenty‐six adult patients (age ≥ 18 years) who tested positive for *H. pylori* by ^13^C‐urea breath test (UBT) were enrolled. Exclusion criteria were: recent use (previous 4 weeks) of antibiotics, bismuth compounds, antisecretory agents, probiotics, or prior *H. pylori* eradication therapy; pregnancy or lactation; gastrointestinal malignancy or ulcer; severe comorbidities; alcohol/drug abuse; or known allergies to pantoprazole or *L. reuteri* . All participants gave written informed consent.

### 2.2 Randomization and Interventions

Patients were randomized 1:1 (using the sealed‐envelope method with Randomizer.org) to one of two regimens for 4 weeks. The *L. reuteri* ‐PPI group received *L. reuteri* DSM 17938 plus 2 × 10^8^ CFU *L. reuteri* ATCC PTA 6475 for 2 × 10^8^ CFU (Gastrus, BioGaia, Stockholm, Sweden), seven times daily, plus pantoprazole 20 mg twice daily. The Placebo‐PPI group received placebo capsules identical to those containing *L. reuteri* (seven times daily, with meals, and at bedtime) plus pantoprazole 20 mg twice daily. Randomization codes were kept sealed until all microbiome data had been collected.

### 2.3 Sample Collection and Outcome Measures

Stool samples were collected at three time points: before treatment (T0), after 4 weeks of therapy (T1), and 1 month post‐therapy (T2). Samples were immediately frozen at −80°C until processing. A ^13^C‐UBT was performed at baseline and 2 months after treatment to confirm *H. pylori* status. The primary outcome variable for the trial was the gut microbiota composition, as determined by metagenomic sequencing of stool samples. Participants who remained *H. pylori* positive at the post‐treatment ^13^C‐UBT were subsequently offered standard antibiotic eradication therapy.

### 2.4 Metagenomic Sequencing and Analysis

Stool samples were thawed and vortexed, and 800 μL were transferred to a ZR BashingBead lysis tube. A positive control of 75 μL of ZymoBIOMICS Community standard in 725 μL DNA/RNA shield and a negative control of 800 μL of DNA/RNA shield were included.

All samples were bead‐beaded in a FastPrep24 5G, followed by centrifugation at 10,000 g for 1 min. Then, 200 μL of the sample was added to a deep‐well plate for purification using a Tecan Fluent, according to the ZymoBIOMICS 96 MagBead DNA protocol. Subsequently, elution was performed with 75 μL of EB buffer (Qiagen). The eluted DNA was stored at −20°C until further analysis.

Fifty nanograms of genomic DNA were used for library preparation with MGI's FS library prep set (MGI, Shenzhen, China) according to the manufacturer's instructions. The quality of the libraries was confirmed using the TapeStation D1000 kit (Agilent, USA), and their quantity was assessed with the QuantIT HighSensitivity dsDNA Assay (Thermofisher, USA) on a Tecan Spark (Tecan, Switzerland). Circularized DNA of equimolarly pooled libraries was prepared using the MGI Easy Circularization kit (MGI Tech). According to the manufacturer's instructions, DNBseq 100 bp paired‐end sequencing was performed using a DNBSEQ‐T7 sequencing instrument (MGI, Shenzhen, China).

Raw reads were quality‐filtered to remove low‐quality and host‐derived sequences. FASTA‐formatted metagenomic files were rarefied to 26,417,664 reads. MetaPhlAn4 software was used for taxonomic profiling. Short DNA reads were assembled into longer contiguous sequences (contigs) to reconstruct genomes. The initial profile comprised 1456 taxa, which were filtered according to the following parameters to remove spurious taxa. After filtering, 495 taxa remained, corresponding to 93% of their taxonomic profile. The diversity of microbial communities was evaluated using metrics including alpha and beta diversity. Taxa were identified that were differentially abundant across sample groups. Non‐metric MultiDimensional Scaling (NMDS) was used for data visualization and to explore relationships between samples based on their microbial composition. Partial Least Squares Discriminant Analysis (PLS‐DA), a machine learning tool used to enhance the signal‐to‐noise ratio, was employed to compare *L. reuteri* ‐PPI with Placebo‐PPI at the three time points. Permutational Multivariate Analysis of Variance (PERMANOVA) test was used to assess whether centroids (group averages in multivariate space) differ significantly between the two groups. All PERMANOVA analyses were performed with Aitchison distance and 999,999 permutations. Significance was assessed at *p* < 0.05 with appropriate multiple‐testing correction where applicable.

### 2.5 Ethical Consideration

The study was approved by the local Institutional Review Board “Comitato Etico ASL n. 1 di Sassari” (Prot. 2198/CE). The study was conducted in accordance with Good Clinical Practice and the Declaration of Helsinki. All patients were informed of the study procedure and potential risks, and they provided written consent. Patients who did not receive immediate eradication therapy as part of the study were assured that they would receive standard‐of‐care treatment after the study period.

## 3 Results

**FIGURE 1:**
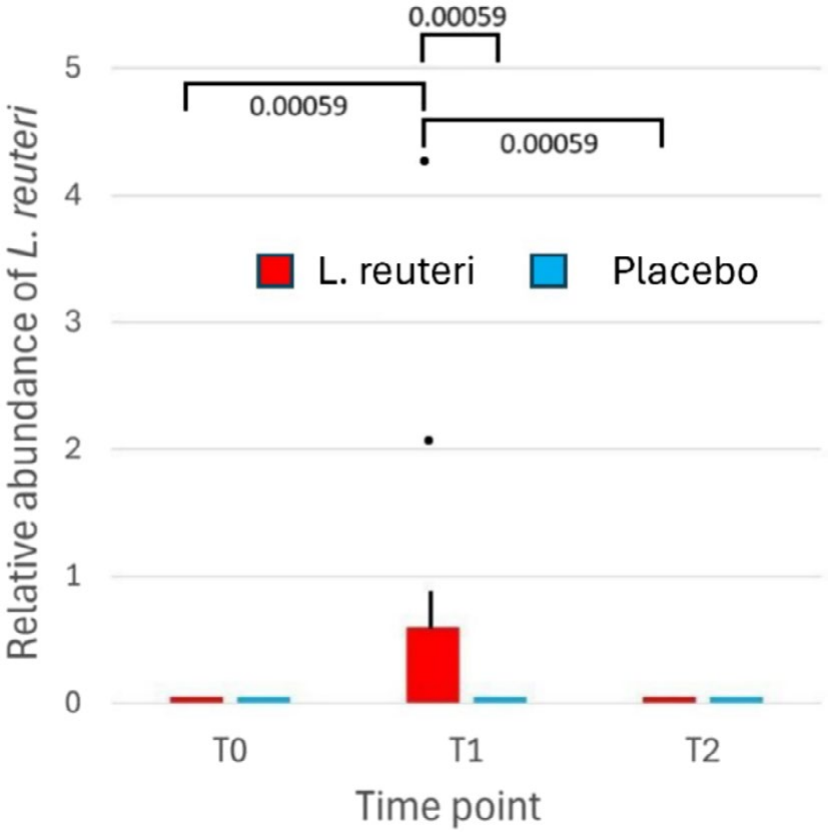
*Lactobacillus reuteri* abundance over time in stool. Relative abundance of *L. reuteri* at baseline (T0), end‐of‐treatment (T1), and 1‐month follow‐up (23) in the *L. reuteri* + PPI vs. Placebo + PPI groups. Boxes show median and interquartile range; *L. reuteri* was detected exclusively in the probiotic group at T1, confirming adherence (Wilcoxon *p* < 0.001).

Table 1 summarizes the patient's characteristics. All 26 patients completed the study without major protocol deviations. Adherence was confirmed by microbiological evidence of *L. reuteri* in stool only in the *L. reuteri* group at T1. Specifically, the relative abundance of *L. reuteri* at T1 was significantly higher in the *L. reuteri* ‐PPI group than in the placebo‐PPI group (Wilcoxon rank‐sum test *p* = 0.00059), reflecting ingestion and gastrointestinal survival of the probiotic capsules. In the *L. reuteri* + PPI group, the abundance of *L. reuteri* rose sharply by T1 (median relative abundance in stool). Then, it declined back to baseline levels by T2 (1 month post‐treatment), indicating that the strain did not persist in the gut long‐term. As expected, no *L. reuteri* was detected in any patient at baseline (T0) or in any of the placebo group samples, as shown in Figure 1.

**TABLE 1:** Baseline characteristics of the two groups.

Characteristics	Treatment group	Placebo group
No. of patients	13	13
Sex (Male/Female)	5/8	4/9
Mean Age (years)	52.3	54.9
Mean Body Mass Index	22.3	23.9
Smoker	2	3

**FIGURE 2:**
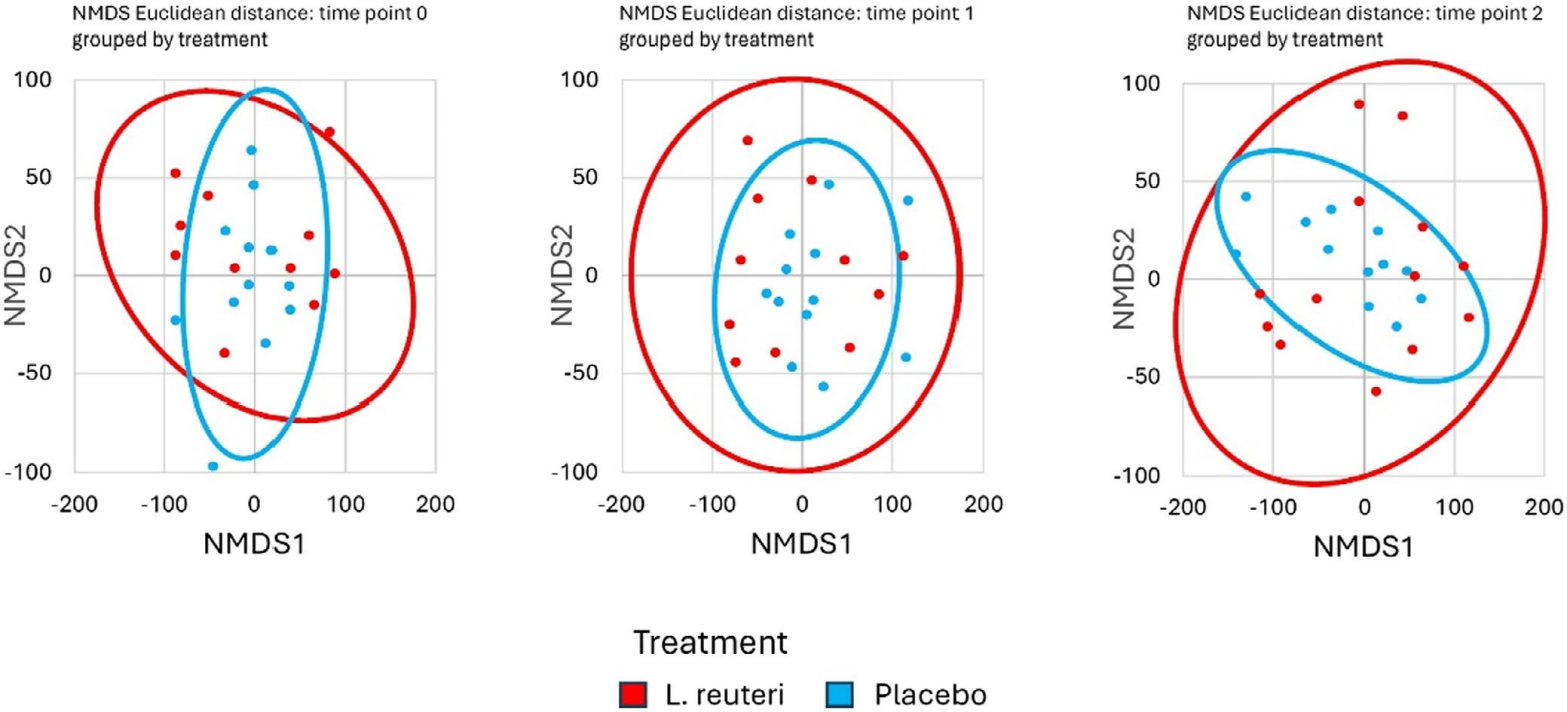
NMDS ordination of gut microbiota composition. Each plot shows samples from *L. reuteri* + PPI (blue) and Placebo + PPI (red) at a given time point (T0, T1, T2). No distinct clustering by treatment is observed at any time point, indicating a high degree of overlap between groups.

**FIGURE 3:**
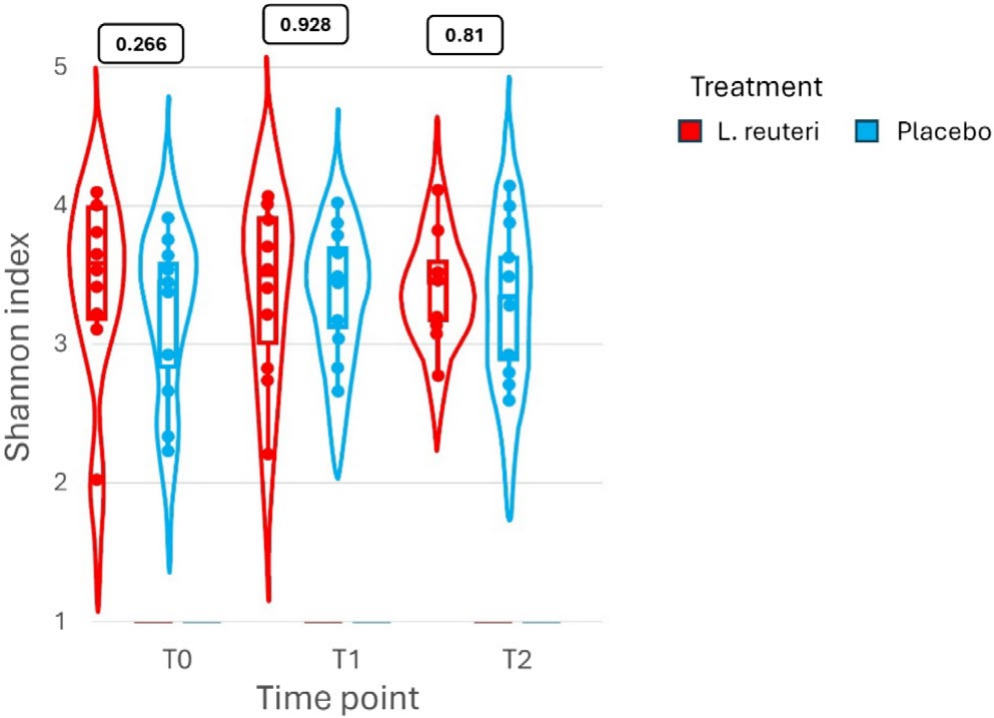
Shannon diversity (alpha‐diversity) in each group over time. Shannon index values for each group (mean ± SD) at T0, T1, and T2. There were no significant differences between the *L. reuteri* vs. Placebo groups at any time (Wilcoxon tests, *p* > 0.05), and no notable changes over time, reflecting stable within‐sample diversity.

Beyond the transient presence of the probiotic strain in the treatment arm, the overall gut microbiota composition showed minimal differences between groups throughout the study. NMDS ordination plots (Figure 2) revealed no distinct clustering by treatment group at any time point, and no apparent separation between baseline (T0) and follow‐up (T2) samples in either group. Inter‐subject variability in microbiome profile was much larger than any effect of the intervention. The Shannon diversity index (alpha diversity) remained stable over time and did not differ significantly between the *L. reuteri* and placebo groups at any visit (*p* > 0.05 by Wilcoxon test at each time point; see Figure 3), indicating that alpha diversity remained comparable regardless of probiotic use. By PERMANOVA analyses, we found no significant statistical differences in community composition between the *L. reuteri* + PPP and placebo + PPI at any single time point (all *p* > 0.4). Likewise, there were no significant longitudinal changes in microbiota composition within either treatment group (baseline vs. T1 vs. T2; all *p* > 0.99 by PERMANOVA). The only significant PERMANOVA result was observed when pooling all samples by treatment group (i.e., *L. reuteri* vs. placebo overall, across all time points), with a small but significant group effect (*R* ^2^ ≈0.03, *p* = 0.0007; Table 2). This overall difference is likely driven by the transient high abundance of *L. reuteri* at T1 in the probiotic group, as no other consistent differences were noted.

**TABLE 2:** PERMANOVA results for beta‐diversity comparisons between groups and across time points.

Comparison made	*R* ^2^‐values	*p*
Placebo‐*L. reuteri*: Time point 0	0.0397	0.6944
Placebo‐*L. reuteri*: Time point 1	0.0447	0.4594
Placebo‐*L. reuteri*: Time point 2	0.0387	0.5901
Placebo‐*L. reuteri*: TP0 vs TP1	0.0186	0.9964
Placebo‐*L. reuteri*: TP0 vs TP2	0.0133	0.9999
Placebo‐*L. reuteri*: TP1 vs TP2	0.0187	0.9953
Placebo‐Placebo: TP0 vs TP1	0.017	0.9969
Placebo‐Placebo: TP0 vs TP2	0.013	0.9993
Placebo‐Placebo: TP1 vs TP2	0.016	0.9964
Placebo‐*L. reuteri*: all time points	0.0313	**0.0007**

**FIGURE 4:**
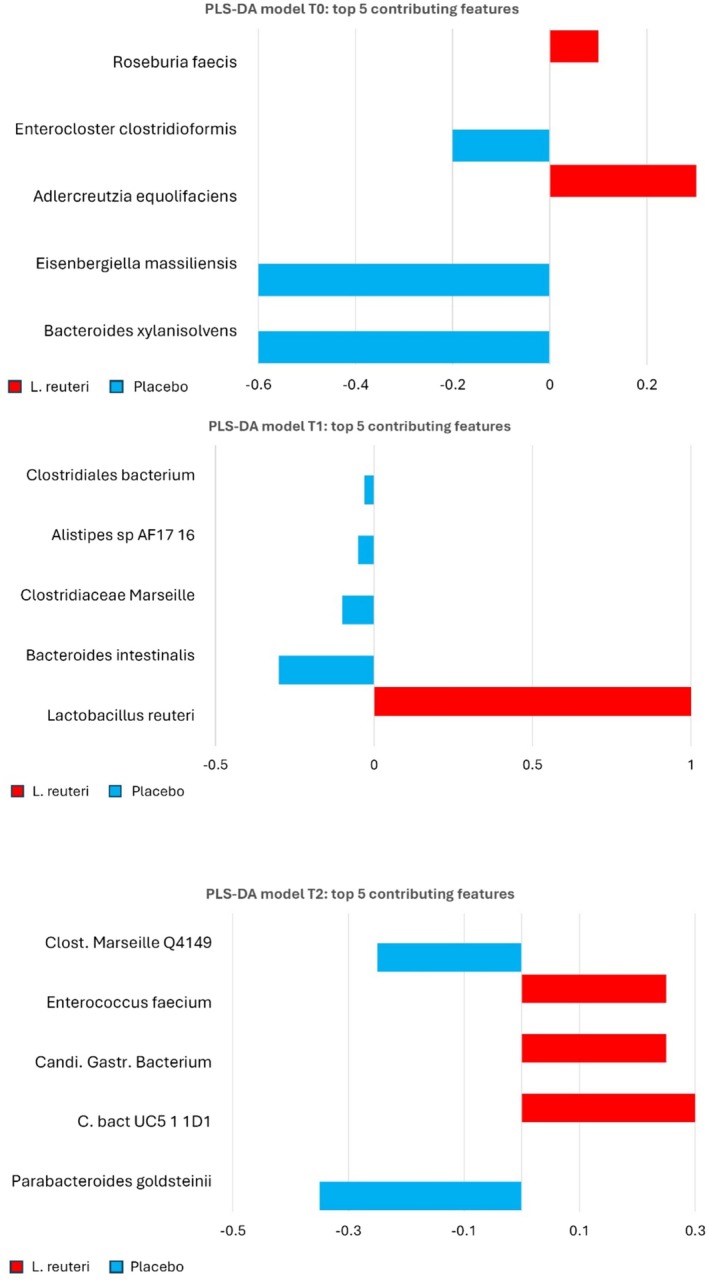
PLS‐DA of microbial profiles. PLS‐DA score plots for T0, T1, and T2 samples, showing the two treatment groups. At T1, samples separate primarily along Component 1 by the presence of *L. reuteri* (blue circles, probiotic group, shift to the right). By T2, this separation disappears, indicating no lasting group difference. (Component variance explained: [X%], [Y%] for comp1, comp2 at each timepoint).

Consistent with these findings, PLS‐DA did not identify any robust or stable taxonomic signature distinguishing the two treatment arms. At T1 (end of treatment), the presence of *L. reuteri* itself was the dominant feature separating groups (as expected, since only the probiotic arm had *L. reuteri* in the microbiota at that point). However, by T2 (1 month later), this signal had disappeared, and the two groups' profiles had become indistinguishable in the PLS‐DA space. No other bacterial taxa showed statistically significant differences in relative abundance between the *L. reuteri‐PPI* and Placebo‐PPI groups at any time point (Figure 4).

Finally, we examined whether any specific taxa were transiently perturbed by the PPI and/or probiotic. Aside from *L. reuteri* in the treated group, no taxa met significance criteria for differential abundance when comparing groups at T1 (after multiple‐testing correction). Similarly, within each group, we did not observe any taxa that changed significantly from baseline to post‐therapy period, supporting the conclusion that the short‐term PPI (with or without *L. reuteri* ) caused minimal disruption to the gut microbiota composition.

## 4 Discussion

In this double‐blind, randomized, placebo‐controlled trial, we investigated the impact of a 4‐week course of pantoprazole, administered either with high‐dose *L. reuteri* supplementation or with placebo, on the gut microbiome of adults infected with *H. pylori* . We found that the gut microbiota exhibited a remarkable resilience to the short‐term intervention. *L. reuteri* was detectable in stool only during the supplementation period and did not persist after probiotic administration ceased. Correspondingly, we observed no significant differences between the probiotic and placebo groups in overall microbial community structure or diversity at any time point, except for the presence of *L. reuteri* itself. Furthermore, the microbiota of both groups remained essentially unchanged from baseline 1 month after therapy, indicating that any transient perturbations during the PPI/probiotic exposure were rapidly reversed upon cessation of treatment.

These findings align with and extend prior observations about microbiome resilience after PPI use. Chronic or high‐dose PPI therapy has been associated with dysbiosis (e.g., lowered diversity and expansion of oral/facultative anaerobes in the gut) in some studies [21, 22, 23]. However, many of those observations come from prolonged PPI use or special populations with additional risk factors. Notably, a recent longitudinal study in healthy adults reported that gut microbiota changes induced by an 8‐week PPI course were largely reversible, with the community tending to return to its pre‐treatment state within weeks after discontinuation of the PPI [29]. Our results are consistent with this pattern of resilience. In our cohort, by 1 month after a 4‐week pantoprazole course, we found no residual differences in microbiome diversity or core composition relative to baseline. In other words, any shift that may have occurred during the PPI exposure had rebounded by the follow‐up. Additionally, the dose of pantoprazole used in our study (20 mg BID) is a moderate acid suppression regimen, and we did not detect notable dysbiotic effects from it over the short term.

Regarding the probiotics, our data align with known characteristics of *L. reuteri* and related probiotic strains. *L. reuteri* secretes antimicrobial compounds (including reuterin, hydrogen peroxide, and lactic acid) that inhibit pathogens and can transiently remodel the gut microbial communities [16]. In clinical studies, oral *L. reuteri* often results in short‐term colonization of the GI tract. For example, one trial showed that *L. reuteri* supplementation significantly increased overall gut microbiota richness, becoming a dominant community member during dosing; however, this effect waned after supplementation ended [30]. We similarly observed that in our *L. reuteri* group, the probiotic organism bloomed to high levels during treatment (accounting for the primary difference between groups at T1) but declined to baseline by 1 month post‐therapy. This transient occupancy likely explains the lack of durable changes to the microbiome. Once supplementation cleared, the endogenous community composition reasserted itself. Notably, we did not detect any other taxa that were significantly altered, suggesting that *L. reuteri* did not competitively exclude or markedly augment other microbes in this context.

Our findings also suggest that co‐administration of *L. reuteri* did not substantially alter the trajectory of post‐PPI microbiota recovery. Some reports have implied that probiotics might mitigate PPI‐related dysbiosis. For example, a pediatric study mentioned earlier found that *L. reuteri* given with PPI reduced the incidence of small intestinal bacterial overgrowth compared to PPI alone [28]. We did not specifically evaluate SIBO; however, our metagenomic analysis did not reveal a significant difference in the overall community composition between the *L. reuteri* and placebo arms. This indicates that, at least in our adult cohort, adding *L. reuteri* did not produce a detectable protective effect on the gut microbiome beyond the inherent resilience it displayed. It is possible that more subtle benefits of the probiotic (e.g., suppression of opportunistic pathogens or functional metabolic effects) occurred but were not captured by our taxonomic analysis. We also acknowledge that effects might differ in other populations (such as children or individuals prone to dysbiosis).

Our study has limitations. The sample size was modest, which may have limited the power to detect very subtle taxonomic shifts. The 1‐month follow‐up period post‐treatment may also be insufficient to capture very delayed or transient rebound effects. For these reasons, we did not conduct complementary functional analyses (e.g., metabolomics), so any subtle shifts in microbial metabolic activity or host–microbe interactions might have gone undetected, despite the stable community composition. We did not include a PPI‐free control group, and the PPI used is of moderate potency (20 mg pantoprazole is roughly equivalent to 9 mg of 40 mg omeprazole). Thus, we cannot directly quantify the effect of the PPI itself, and our primary question was whether adding *L. reuteri* would change the outcome relative to PPI alone; we found no evidence of a significant difference in that regard. Additionally, our analysis focused on the fecal microbiota, rather than the gastric mucosal microbiota; therefore, we cannot comment on potential probiotic effects within the stomach or *H. pylori* eradication rates. Neither question was the primary focus of this study. However, it is well established that probiotic supplementation during *H. pylori* treatment can confer modest clinical benefits, including slightly higher eradication success and reduced therapy‐related side effects. Our trial was not designed to evaluate clinical outcomes; therefore, our finding of microbiome stability should be interpreted in conjunction with the known clinical advantages of probiotics. However, the absence of microbiota disruption observed here does not contradict the evidence that probiotics can improve patient outcomes as adjuncts to *H. pylori* therapy.

In conclusion, our results provide a reassuring message: 4 weeks of the PPI, pantoprazole, combined with *L. reuteri* did not induce significant adverse changes in the gut microbiota of *H. pylori* ‐infected adults. Supplemental *L. reuteri* transiently increased the detectable *L. reuteri* population in stool during therapy, but failed to colonize for long term the gut. No other significant microbiome shifts were observed, and both alpha‐ and beta‐diversity metrics remained stable across groups and over time. These findings suggest that the gut microbiota is resilient and returns to baseline after a short‐term PPI challenge, even when a high‐dose probiotic is co‐administered. In essence, the use of probiotics during a brief PPI treatment appeared to have a microbiologically neutral effect. Our work thereby isolates the effect of acid suppression on the microbiome, indicating that a short, moderate‐dose PPI course causes minimal disruption. Future studies with larger cohorts, longer probiotic regimens, the inclusion of a PPI‐free control group, and the use of highly potent acid suppressants or different patient populations (e.g., those prone to dysbiosis) will be valuable for fully elucidating the interactions between acid suppression, probiotics, and the intestinal microbiota. Additionally, incorporating multi‐omics (metabolomic, metatranscriptomic) analyses in future research could reveal functional microbiome changes that are not evident from taxonomic profiles alone.

## Conflicts of Interest

The authors declare no conflicts of interest.

## Data Availability

The data that support the findings of this study are available on request from the corresponding author. The data are not publicly available due to privacy or ethical restrictions.
